# Natural products modulate NLRP3 in ulcerative colitis

**DOI:** 10.3389/fphar.2023.1265825

**Published:** 2023-10-02

**Authors:** Jia-Chen Xue, Shuo Yuan, Xiao-Ting Hou, Huan Meng, Bao-Hong Liu, Wen-Wen Cheng, Ming Zhao, Hong-Ben Li, Xue-Fen Guo, Chang Di, Min-Jie Li, Qing-Gao Zhang

**Affiliations:** ^1^ Department of Nuclear Medicine, Affiliated Zhongshan Hospital of Dalian University, Dalian, Liaoning, China; ^2^ Chronic Disease Research Center, Medical College, Dalian University, Dalian, Liaoning, China; ^3^ Department of Immunology and Pathogenic Biology, Yanbian University College of Basic Medicine, Yanji, Jilin, China; ^4^ Key Laboratory of Natural Medicines of the Changbai Mountain, Ministry of Education, College of Pharmacy, Yanbian University, Yanji, Jilin, China

**Keywords:** Chinese medicine, inflammatory bowel disease, NLRP3, traditional natural products, ulcerative colitis

## Abstract

Ulcerative colitis (UC) is a clinically common, progressive, devastating, chronic inflammatory disease of the intestine that is recurrent and difficult to treat. Nod-like receptor protein 3 (NLRP3) is a protein complex composed of multiple proteins whose formation activates cysteine aspartate protease-1 (caspase-1) to induce the maturation and secretion of inflammatory mediators such as interleukin (IL)-1β and IL-18, promoting the development of inflammatory responses. Recent studies have shown that NLRP3 is associated with UC susceptibility, and that it maintains a stable intestinal environment by responding to a wide range of pathogenic microorganisms. The mainstay of treatment for UC is to control inflammation and relieve symptoms. Despite a certain curative effect, there are problems such as easy recurrence after drug withdrawal and many side effects associated with long-term medication. NLRP3 serves as a core link in the inflammatory response. If the relationship between NLRP3 and gut microbes and inflammation-associated factors can be analyzed concerning its related inflammatory signaling pathways, its expression status as well as specific mechanism in the course of IBD can be elucidated and further considered for clinical diagnosis and treatment of IBD, it is expected that the development of lead compounds targeting the NLRP3 inflammasome can be developed for the treatment of IBD. Research into the prevention and treatment of UC, which has become a hotbed of research in recent years, has shown that natural products are rich in therapeutic means, and multi-targets, with fewer adverse effects. Natural products have shown promise in treating UC in numerous basic and clinical trials over the past few years. This paper describes the regulatory role of the NLRP3 inflammasome in UC and the mechanism of recent natural products targeting NLRP3 against UC, which provides a reference for the clinical treatment of this disease.

## Graphical Abstract

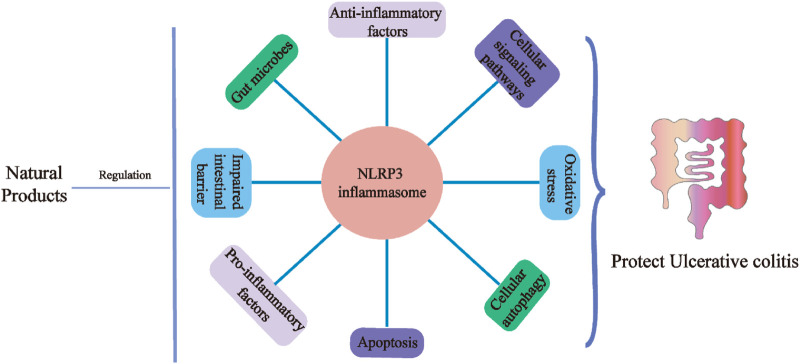

## 1 Introduction

Ulcerative colitis (UC) is a type of inflammatory bowel disease (IBD) that is characterized by persistent or recurrent immune activation and intestinal inflammation with recurrent clinical manifestations such as abdominal pain, bloating, and diarrhea (Caron et al., 2022). In the 20th century, UC occurred mainly in Europe and the United States, and as time progressed, the incidence of UC accelerated in newly industrialized countries in Asia, South America, and Africa, affecting more than 6.8 million people worldwide (Paik, 2022). The intestinal mucosal layer is invaded by a large number of neutrophils, lymphocytes, and monocytes, causing a strong inflammatory response, and damage to the mucosal barrier affects flora and immunity (Buie et al., 2022; Guo et al., 2022). NOD-like receptors (NLR) are the prototypical cytoplasmic receptors, and NLRP3 (NOD-like receptor 3), a member of the NLR family, is rapidly becoming a crucial regulator of intestinal homeostasis (Liu et al., 2022). NLRP3 responds to a range of pathogenic microorganisms and danger signals to maintain body equilibrium, which can activate pro-caspase-1 to caspase-1. Thereby, NLRP3 releasing the pro-inflammatory cytokines interleukin (IL)-1β and IL-18, results in inflammatory cell death, which is closely related to the pathogenesis of UC(Xue et al., 2022). NLRP3 deletion and intestinal epithelial proliferation when moderately activated can directly affect gut microbes, but overactivated NLRP3 directly triggers intestinal inflammation.

Traditional Chinese medicine and its natural active ingredients, such as extracts, have attracted much attention for their distinct theoretical framework, multi-target and multi-pathway, mild action, and low toxicity, as well as for their increasingly noteworthy effectiveness in preventing and treating complex diseases caused by multiple etiologies. The extraction of active compounds from natural products or herbal remedies to treat UC has gained popularity in recent years (Li et al., 2018). This article provides a rationale for developing therapies that target natural products. These natural products target NLRP3 and may reduce the intestinal inflammatory response in UC patients.

## 2 Treatment of UC

UC is a chronic inflammatory bowel disease. It has a long course of the disease and lacks effective treatment and management, which is difficult to cure (Burri et al., 2020). Although drug treatment can alleviate clinical symptoms, the patient cannot be fully recovered. Therefore, UC is considered to be an incurable disease. At present, the etiology and pathogenesis of UC are still unclear, and there is still a lack of specific targeted therapeutic drugs (Jeong et al., 2019). The commonly used therapeutic drugs for UC are mainly aminosalicylic acid, glucocorticoids, immunosuppressive agents, non-steroidal anti-inflammatory drugs, and intestinal microecological preparations. Surgical treatment can be considered in cases of serious diseases that cannot be treated medically.

Finding safe and effective drugs is a significant challenge for the clinical treatment of UC(Ungaro et al., 2017). In comparison with salazosulfapyridine, mesalamine and olanzapine are better tolerated by patients with UC(Lee et al., 2020). The use of high-dose mesalazine in patients with severe UC is more effective than low-dose mesalazine, but high-dose drug application can lead to dose-dependent toxicity (Ungaro et al., 2017). In addition, patients must also deal with the high costs of UC treatment. The cost of long-term drug treatment increases the patient’s financial strain and reduces their quality of life. The therapeutic effect of biological agents such as monoclonal antibodies on moderate and severe UC is significantly better than that of non-biological agents (i.e., aminosalicylic acid preparations) despite the higher cost of treatment. Mesalazine is the first-line drug for IBD clinical treatment, but its cost is higher than sulfasalazine. The cost of drugs and treatment efficacy should be considered simultaneously when treating IBD patients. There is a major clinical challenge in finding effective low-cost treatment options for each patient (Stawowczyk and Kawalec, 2018).

## 3 Overview of NLRP3 inflammasome

Known as inflammasomes, these proteins detect intracellular damage-associated molecular patterns or pathogen-associated molecular patterns. Additionally, IL-1β and IL-18 are released after being processed, matured, and released by this protein complex. The body’s immune cells play an essential role in resisting pathogen infection and stress injury, but excessive activation can lead to inflammatory effects and organ damage (Theodoropoulou et al., 2022).

### 3.1 NLRP3 inflammasome structure and activation

The Apoptosis-Associated Speck-Like Protein (ASC), and the aforementioned three structural domains of caspase-1 make up the high molecular weight multiprotein complexes known as NLRP3 inflammasomes, which are primarily expressed in neutrophils, macrophages, lymphocytes, monocytes, osteoblasts, and dendritic cells (Liao et al., 2021). The activation of the NLRP3 inflammasome has been associated with several internal and external signals, including cholesterol, lipopolysaccharides, calcium pyrophosphate, silica, sodium urate, palmitate, and pathogen-associated molecular patterns. Many damage-associated molecular patterns activate this inflammasome, including hyperglycemia, hypercalcemia, hypokalemia, ATP, reactive oxygen species (ROS), and cathepsin B (Gold and El Khoury, 2015). The NLRP3 inflammasome is also activated by sodium channels, specifically dysregulated epithelial sodium channels (Scambler et al., 2019).

**FIGURE 1:**
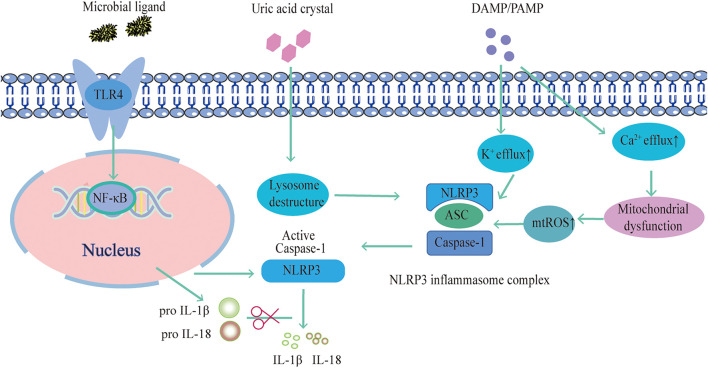
NLRP3 inflammasome activation pathway.

Researchers have examined the mechanism of the NLRP3 inflammasome in search of potential targets for pharmacological intervention in associated disorders. There are four primary ideas that have so far been accepted. Firstly, NLRP3 is activated by intracellular K^+^ efflux. Bacterial toxins such as Nigericin can cause membrane holes that cause intracellular K^+^ efflux or activate P2X7 receptors (Gong et al., 2015). Besides, studies have shown that inhibition of ROS production or expression of the mitochondrial protein VDAC can prevent NLRP3 inflammasome activation (Nguyen et al., 2022). According to Wang et al., the ROS inhibitor acetylcysteine significantly reduced mitochondrial damage and ROS production, which is fundamental for the activation of NLRP3. By activating NLRP3, NLRP3 inflammasome component proteins move about the mitochondria. As a result, mitochondrial damage and ROS production are crucial for NLRP3 activation (Wang H. et al., 2017). In addition, Ca2+ influx affects NLRP3 inflammasome activation interestingly. The Ca^2+^ extracellularly reduces the negative transmembrane potential in mitochondria and releases it into the cytoplasm during endoplasmic reticulum stress. Additionally, intracellular Ca^2+^ negative feedback encourages the association of NLRP3 and ASC histone proteins, and ASC oligomerization mediates NLRP3 inflammasome activation (Lin et al., 2022). Also, inflammasome activation is facilitated by the endocytosis of granular substances by macrophages, such as cholesterol crystals and SiO_2_, which causes lysosomal fragmentation and disruption of histone proteases. Cathepsin B is released as a result of this fragmentation, facilitating NLRP3 inflammasome activation (Shao et al., 2022).. (Figure 1.)

### 3.2 Negative regulation mechanism of NLRP3 inflammasome

#### 3.2.1 Negative regulation of NLRP3 inflammasome expression

The activity of nuclear factor kappa-B (NF-κB) can be inhibited by estrogen and the zinc finger protein. In addition, zinc finger protein and aromatic hydrocarbon receptors can bind to exogenous chemical response elements on NLRP3 (Hajikhezri et al., 2020). Endogenous miR-233, which is highly expressed in the myeloid lineage, and mir-BART15, secreted by B cells after EB virus infection, can bind to the 3′untranslated end of NLRP3 mRNA, making it a target for degradation, accelerating its degradation, and negatively regulating NLRP3 inflammasome at the translational level (Yang et al., 2015).

#### 3.2.2 Negative regulation of NLRP3 inflammasome assembly

Depending on the structure of the inflammasome, the central molecule usually contains the pyrin domain-caspase activating and recruitment domain region (Martinon et al., 2009; Davis et al., 2011). Proteins in this domain competitively bind to these molecules, thereby inhibiting the assembly of complexes. NLRP3 complex assembly is negatively regulated by proteins with a pyrin domain, which interferes with the signal transduction between ASC and NLRP3 (Martinon et al., 2009). Another class contains caspase recruitment domains such as only proteins, caspase-12, and other molecules, which inhibit NLRP3 activation by competitively binding to ASC with caspase-1. In addition, the protease inhibitor PI-9 can suppress the caspase-1 activation by competitively binding to the active site of caspase-1 (Kannan-Thulasiraman and Shapiro, 2002). The small heterodimer partner is recruited to mitochondria upon activation to bind NLRP3 competitively with ASC and inhibit NLRP3 inflammasome complex assembly (Yuk et al., 2016).

#### 3.2.3 Negative regulation of NLRP3 inflammasome activation

NLRP3 inflammasome activation methods vary, making the negative control of this pathway’s activation even more difficult to understand. Negative regulation of the NLRP3 inflammasome activation pathway is more complex due to the diversity of activation mechanisms. The cells can reduce their content of damaged mitochondria and ROS by activating the autophagy pathway under stress, thus negatively regulating NLRP3 activity (Zhou et al., 2011). Some substances, such as *in vivo* metabolites such as β-hydroxybutyric acid, can prevent intracellular K^+^ efflux to reduce ASC oligomerization and spot formation, negative regulation of NLRP3 inflammasome (Rayamajhi and Miao, 2013). In some autoimmune diseases, phospholipase C gamma 2 mutations enhance phospholipase Cγ2 activity, promote intracellular Ca2+ release from the endoplasmic reticulum, and activate the NLRP3 inflammasome (Chae et al., 2015).

#### 3.2.4 Negative regulation mechanism of NLRP3 induced by autophagy

Autophagy, which involves the breakdown and recycling of damaged cells, tissues, and organs, may intensify the inflammatory response (Morimoto and Cuervo, 2009). In autophagy, damaged cell tissues and organs are degraded and recycled (Basisty et al., 2018). Inflammatory reactions can be further exacerbated by the loss of autophagy-related proteins. Stress can inhibit mitochondrial damage and reduce ROS production through autophagy, which inhibits NLRP3 activation (Brookes et al., 2004). Nakahira et al. found that the loss of autophagy protein microtubule-associated protein light chain 3B (LC3B) and Beclin 2 expression was silenced, which activated caspase-1 and caused high expression of IL-18 and IL-1β, suggesting that the possible mechanism is impaired autophagy and mitochondrial dysfunction further activates NLRP3 inflammasome (Nakahira et al., 2011). Zhong et al. reported that NF-κB activation induced by LPS may lead to a further increase in p62/Sequestosome 1 expression, suggesting mitochondrial autophagy can negatively control p62/Sequestosome 1 expression (Zhong et al., 2016). The above shows that NLRP3 inflammasomes can be negatively regulated by mediating mitophagy.

## 4 NLRP3 and UC

### 4.1 The mechanism of NLRP3 involved in UC

UC is a disease characterized by chronic persistent intestinal inflammation caused by gastrointestinal mucosal immune dysfunction (Wang Z. et al., 2022). A large number of immune cells can be provided in the gastrointestinal mucosa to prevent infection with toxins and potential pathogens in the environment. Increasing evidence suggests that innate immune recognition on the mucosal surface, especially in the intestine, is an imperative component of intestinal homeostasis (Verma et al., 2022). It has been further highlighted that the role of the NLRP3 inflammasome is not only as an influential mediator of host defense but also as an effective regulator of homeostasis in the gut by controlling the integrity of intestinal epithelial cells, thereby regulating the immune response of the gut to the microbiota (Li et al., 2022). However, reports on NLRP3’s role in UC remain controversial. NLRP3 inflammasomes may have two aspects in UC. On the one hand, inflammasomes can enhance inflammation and aggravate colon injury. On the other hand, inflammasomes can improve UC progression.

The NLRP3 protein plays a critical role in maintaining intestinal homeostasis and identifying intestinal parasites. It has been found that the NLRP3 inflammasome plays an important role in the pathogenesis of UC(Larabi et al., 2020). Zaki et al. constructed a mouse UC model with 3% dextran sulfate sodium salt (DSS) to evaluate the intestinal homeostasis of NLRP3 gene deletion and wild-type mice. The results showed that NLRP3 gene deletion mice were more susceptible to UC, and macrophages isolated from NLRP3 gene deletion mice had no immune response to bacterial muramyl dipeptide (Zaki et al., 2010). In mice with NLRP3 deficiencies, 2.5% DSS or (2,4,6-trinitro-Benzenesulfonic acid) TNBS enema caused more severe colonic inflammation (Lazaridis et al., 2017). However, the role of NLRP3 in UC is also controversial. Kanneganti et al. (Kanneganti, 2017) reported that the susceptibility of NLRP3 gene-deficient mice to 2% DSS-induced UC was significantly reduced. It has also been found that caspase-1 gene-deficient mice were given DSS induction. The symptoms of UC are alleviated, and the mechanism may be related to the decreased secretion of pro-inflammatory factors such as IL-18 and IL-1β in colonic mucosa (Wang et al., 2018).

The mechanism by which NLRP3 participates in UC is very complex, and a variety of cytokines are involved. Wang et al. (Wang et al., 2018) reported that DSS destroys lysosomes *in vivo* and activates NLRP3 inflammasome, promoting the release of IL-18 and IL-1β, thereby inhibiting UC induced by DSS. At the same time, it was found that NLRP3 inflammasome had effects on TNFα, IL-10, interferon (IFN)-γ, transforming growth factor (TGF) -β, myeloperoxidase (MPO), β-defensin and intestinal flora. Further research is needed to determine its specific mechanism of action and to guide clinical treatment. Therefore, further studies are needed to determine the exact role of the NLRP3 inflammasome in UC.

### 4.2 NLRP3-related targeted clinical studies in UC

In recent years, research on the mechanism of action and targeted therapy of NLRP3 in UC has become more and more in-depth. The research on the activation and regulation mechanism of NLRP3 inflammasome mainly focuses on immune cells and animal models. The NLRP3 inflammasome clinical research has also made some progress. In medical practice, IBD is mainly treated with antibodies or antagonists that inhibit NLRP3 activation, including IL-1β receptor blocker Rilonacept, IL-18 blocker GSK1070806, NLRP3-related ATPase inhibitor Bay 11-7082, and caspase-1 inhibitor eugenol, some of which have been or will soon enter clinical trials (Ozaki et al., 2015). There are also treatments that indirectly inhibit NLRP3 activation, such as inhibiting the pro-caspase1 cleavage pathway, blocking the NF-κB into the nucleus, and reducing mitochondrial damage. In addition, studies have used synthetic NLRP3 molecular inhibitors in animal models to evaluate therapeutic effects (Du et al., 2017).

## 5 Regulation of NLRP3 in UC by natural products

The control of NLRP3 inflammasome assembly by natural products has been shown to ameliorate UC illness in recent research. This article summarizes studies on the creation of natural UC extracts based on the NLRP3 inflammasome over the past 10 years.

### 5.1 Polyphenols

Traditional medicine has utilized phenolic chemicals in foods and beverages for thousands of years, including fruits, vegetables, beverages, herbs, and spices^(^ Yu et al., 2022). Intestinal inflammation and polyphenols have drawn more attention in recent years (Table 1).

**TABLE 1:** Natural products of Phenols modulate NLRP3 protection in UC.

Natural products	Phenols		
Ingredient/Dose	Pineapple Leaf Phenols 200 mg/kg; 20 μg/mL	Rosmarinic acid 5,10,20 mg/kg	Resveratrol 50,100,200 mg/kg
Chemical name		(2R)-3-(3,4-Dihydroxyphenyl)-2-(2E)-3-(3,4-dihydroxyphenyl)prop-2-enoyl]oxy propanoic acid	3,5,4′-trihydroxy-trans-stilbene
Chemical formula		C_18_H_16_O_8_	C_14_H_12_O_3_
Type of cells	LPS-induced RAW 264.7 cells; DSS-induced Caco-2 cells		
Model of UC	DSS-induced UC mice	DSS-induced UC mice	Radiation-induced IBD mice
Route of administration	Oral	Oral	Oral
Mechanism	Inhibit NF-κB activation and expression of pro-IL-1β; ZO-1, occludin, and claudin 1↑	Upregulate NRF2/HO-1 expression; Reduction of NLRP3, ASC, cleaved caspase-1 expression	NLRP3, IL-1β, TNF-α↓
References	Chen et al. (2021)	Marinho et al. (2021)	Sun et al. (2020)

#### 5.1.1 Pineapple leaf phenols (PLPs)

Pineapple leaf polyphenols, which are organic phenol extracts from pineapple leaves, have lipid-regulating, antioxidant, and anti-diabetic properties (Xie et al., 2014). NF-κB plays an important role in many physiological processes, including inflammation. NF-κB is activated when lymphocytes are activated and differentiated, as well as when innate cells produce pro-inflammatory cytokines. During the inflammasome activation process, NF-κB initiates the transcription of NLRP3 and Pro-IL-1β components (Xu et al., 2022). The PLPs inhibit both NF-κB activation and proinflammatory molecule production such as pro-IL-1β, thus reducing the inflammatory response in mice colonic caused by DSS(Chen et al., 2021). As opposed to other secreted proteins, IL-1β normally requires secondary proteolytic cleavage by the NLRP3 inflammasome to activate and release. By preserving epithelial integrity, PLPs also prevent acute colitis caused by DSS.

#### 5.1.2 Rosmarinic acid (RA)

RA(C_18_H_16_O_8_) is abundant in plants of the *Lamiaceae* and *Lithaceae* families. It is known to have many biological effects, including antiviral, anti-inflammatory, and antibacterial (Mishra et al., 2021). By regulating the NLRP3 inflammasome and restoring the nuclear factor-erythroid 2-related factor 2 (Nrf2)/heme oxygenase 1 (HO-1) signaling pathway, RA nanovesicles may protect the colonic mucosa from damage caused by DSS(Marinho et al., 2021). Consequently, this formulation may offer a cutting-edge nutraceutical approach to treating UC in the oral cavity.

#### 5.1.3 Resveratrol

There is a natural non-flavonoid polyphenol in grapes and wine called resveratrol (C_14_H_12_O_3_). The use of resveratrol (and its derivatives) for the treatment and prevention of numerous chronic diseases has shown promise, such as IBD and diabetes (Kitada et al., 2011). Several lines of evidence suggest that resveratrol reduces intestinal inflammation and damage (Dong et al., 2013). Research has shown that resveratrol inhibits the RAC-alpha serine/threonine-protein kinase (AKT1) and NLRP3 inflammasome in cardiomyocytes caused by acute sympathetic stimulation (Wang et al., 2022). According to the research in the animal model of radiation-induced IBD, resveratrol inhibits the expression of IL-1β and the NLRP3 inflammasome, which in turn reduces the expression of IL-1β and its synthesis and secretion (Sun et al., 2020). Resveratrol upregulated HO-1 to protect CaCO_2_ from H_2_O_2_-induced oxidative damage and elevated epithelial expression of Occluding and zonula occludens (ZO)-1 in a dose-dependent manner (Wu et al., 2016). Resveratrol, however, also had effects on CaCO_2_ cells that inhibited growth and stopped the cell cycle (Liu et al., 2014).

### 5.2 Flavonoid

A wide range of fruits, vegetables, cereals, and plants contain flavonoids, which have antioxidant, anti-inflammatory, anti-mutagenic, and neuroprotective properties. The benefits of flavonoids on the gastrointestinal tract have been demonstrated in the past 10 years by growing scientific data (Xue et al., 2023).. (Table 2)

**TABLE 2:** Natural products of Flavonoids modulate NLRP3 protection in UC.

Natural products	Flavonoids							
Ingredient/Dose	Baicalein 20 mg/kg; 5–200 µm	TFGU 135 mg/kg	Lonicerin 3, 10, and 30 mg/kg	Formononetin 25, 50, and 100 mg/kg	Oroxindin 12.5, 25, 50 mg/kg; 12.5, 25, and 50 μM	Alpinetin 25, 50, and 100 mg/kg; 50, 100, 200 μg/mL	Procyanidin 10, 20, 40 mg/kg	Kaempferol 50 mg/kg
Chemical name	5,6,7-trihydroxyflavone		3′,4′,5-Trihydroxy-7-[α-L-rhamnopyranosyl-(1→2)-β-D-glucopyranosyloxy]flavone	7-Hydroxy-4′-methoxyisoflavone	5-Hydroxy-8-methoxy-4-oxoflav-2-en-7-yl β-D-glucopyranosiduronic acid	(2*S*)-7-Hydroxy-5-methoxyflavan-4-one		3,4′,5,7-Tetrahydroxyflavone
Chemical formula	C_15_H_10_O_5_		C_27_H_30_O_15_	C_16_H_12_O_4_	C_22_H_20_O_11_	C_16_H_14_O_4_		C_15_H_10_O_6_
Type of cells	LPS-induced RAW 264.7 cells		LPS-induced BMDMs and THP-1 cells		LPS-induced THP-1 cells	LPS-induced THP-1 cells	LPS-induced THP-1 cells	
Model of UC	TNBS-induced colitis mice	Irinotecan induced colitis mice	DSS-induced UC mice	DSS-induced UC mice	DSS-induced UC mice	DSS-induced UC mice	DSS-induced UC mice	DSS-induced UC mice
Route of administration	Oral	Intraperitoneal injection	Oral	Oral	Oral	Intraperitoneal injection	Oral	Oral
Mechanism	iNOS, ICAM-1, MCP-1, COX-2, TNF-α,IL-1β, TLR4, MyD88, NF-κB p65, p-p65, IκBα, p-IκBα, p-38, p-p38, NLRP3, ASC, caspase-1↓	TNF-α, IL-6 IL-1β, NLRP3, caspase-1↓ Regulating intestinal flora	IL-1β, IL-18, NLRP3, caspase-1↓	IL-1β, IL-18, NLRP3, caspase-1↓	IL-1β, IL-18, NLRP3, caspase-1↓ TXNIP↑	IL-1β, IL-18, ASC, NLRP3, caspase-1, TLR4, p-P65, p-IκB↓	MMP9, IL-1β, IL-18, ASC, NLRP3, caspase-1↓	ZO-1, occludin, and claudin-1, IL-1b, IL-6, and TNF-a, NLRP3, caspase-1↓ TLR4-NF-κB signaling↓
References	Luo et al. (2017)	Yue et al. (2021)	Lv et al. (2021)	Wu et al. (2018)	Liu et al. (2020)	He et al. (2016)	Chen et al. (2017)	Qu et al. (2021)

#### 5.2.1 Baicalein

Traditional Chinese herbs such as *Scutellaria baicalensis Georgi* contain a bioactive flavonoid called baicalein (C_15_H_10_O_5_), a flavonoid with bioactive properties (Kim et al., 2013). It was found that the TLR4/MyD88 pathway was significantly involved in the TNBS-induced production of pro-inflammatory cytokines and mediators, as well as downstream signaling molecules NF-κB and MAPKs, while NLRP3 was necessary for IL-1β release. The TLR4/MyD88 signaling cascades (NF-κB and MAPK) in lipopolysaccharide (LPS)-stimulated macrophages were downregulated by baicalein. The binding of baicalein to the hydrophobic area of the MD-2 pocket prevented the upstream development of the LPS-induced MD-2/TLR4 complex. Further, baicalein dose-dependently reduced downstream IL-1 expression and NLRP3 inflammasome activation. Baicalein suppressed TNBS-induced colitis, at least in part, by inhibiting TLR4/MyD88 signaling and inactivating the NLRP3 inflammasome (Luo et al., 2017).

#### 5.2.2 Total flavonoids of Glycyrrhiza uralensis (TFGU)

*Glycyrrhiza uralensis Fisch* is a natural sweetener and herbal remedy for inflammatory illnesses (Yang et al., 2017). TFGU cures UC in mice by acting as an antioxidant through the Nrf2 pathway and as an anti-inflammatory through the NF-κB pathway. TFGU contains liquiditigenin, isoliquiritigenin, liquiritin, and glycyrrhizin, which are believed to be the main active ingredients of *G. uralensis* (Liu et al., 2018). The Toll-like receptor 4/myeloid differentiation protein 2 complex may be altered by isoliquiritigenin and glycyrrhizin at the receptor level, suppressing the signaling cascade and cytokine production. Additionally, liquiritigenin, isoliquiritigenin, and isoliquiritin may suppress the inhibitor of NF-κB (IκB) degradation and mediate anti-inflammatory responses induced by LPS(Cui et al., 2017). It is significant that isoliquiritigenin is predominantly found in the gastrointestinal tract and has the potential to reduce the severity of dextran sulfate sodium-induced colitis by inhibiting the MAPK pathway (Choi et al., 2016) and preventing colitis-related tumors by preventing the polarization of macrophage M2 by prostaglandin E2 and interleukin-6 (IL-6). As uric acid activates the NLRP3 inflammasome and releases inflammatory mediators such as IL-1β and IL-18, it contributes to inflammatory diseases (Martinon et al., 2006). Increased uric acid produced by an inflammatory reaction in the colon may worsen intestinal illness. When IBD mice were treated with uric acid alone, the condition was aggravated, and intestinal permeability was raised (Chiaro et al., 2017). This study found that TFGU suppressed the activation of the NLRP3 inflammasome in mice with colitis caused by irinotecan. When TFGU therapy was administered to colitis animals, purine metabolism was downregulated, which resulted in a significant decrease in fecal uric acid. As a result of these results, it appears that TFGU’s therapeutic action on irinotecan-induced gastrointestinal damage relies upon a reduction in fecal uric acid and suppression of the NLRP3 inflammasome (Yue et al., 2021).

#### 5.2.3 Lonicerin

Lonicerin (C_27_H_30_O_15_), a flavonoid glycoside found in the flowers of *Lonicera japonica Thunb.*, was commonly mentioned in ancient East-Asian prescriptions for treating inflammatory and infectious diseases (Shang et al., 2011). Several studies have shown that lonicerin has anti-inflammatory and immunomodulatory properties (Lee et al., 1995). Lonicerin increases autophagy to inactivate the NLRP3 inflammasome. According to a study, Lonicerin targets enhancers of zeste homolog 2 to reduce the symptoms of UC in mice by inactivating the NLRP3 inflammasome (Lv et al., 2021). By encouraging NLRP3 degradation to prevent NLRP3 inflammasome assembly, this work demonstrated that lonicerin selectively suppressed NLRP3 inflammasome activation in colonic macrophages. Subsequent research demonstrated that lonicerin improved autophagy to inactivate NLRP3 inflammasome.

#### 5.2.4 Formononetin

Formononetin (C_16_H_12_O_4_) is an isoflavone compound found in many natural plants. Astragalus, for example, contains this compound as one of its main biologically active components (Machado Dutra et al., 2021). Over 2000 years ago, astragalus was used in China to cure diabetes. Formononetin has been shown to have anti-inflammatory, antioxidative, antitumor, and promoting apoptosis properties in recent studies (Ma et al., 2013; Li et al., 2014; Lima Cavendish et al., 2015). It has been reported that formononetin reduces inflammation in rats with peritonitis and protects mice from acute lung damage caused by LPS (Ma et al., 2013). *In vitro*, formononetin reduced acute damage caused to colonic cells by TNF-α by selectively boosting tight junction protein expression. Meanwhile, formononetin reduced NLRP3 pathway protein levels *in vivo* and *in vitro* (NLRP3, ASC, IL-1β) (Wu et al., 2018).

#### 5.2.5 Oroxindin

The chemical molecule oroxindin (C_22_H_20_O_11_) is a flavone and a phenolic compound. Specifically, it is a wogonoside, or wogonin glucuronide, which was isolated from *Holmskioldia sanguinea*, O*roxylum indicum*, and *Bacopa monnieri* (all Bignoniaceae) (Fong et al., 2019). It was discovered that Huang-Qin (one of the key ingredients of Huang-Qin-Tang) contains a natural bioflavonoid called oroxindin. Studies have shown that oroxindin has anti-inflammatory, anti-tumor, and anti-oxidant properties (Wang et al., 2017). Researchers found that oroxindin reduces inflammation in the colon by inhibiting the formation and activation of the NLRP3 inflammasome, which is connected to its inhibitory effects on the TXNIP-dependent NF-κB signaling pathway (Liu et al., 2020).

#### 5.2.6 Alpinetin

Alpinetin (C_16_H_14_O_4_) is a flavonoid found in ginger as well as large amounts of cardamom, turmeric, and tulip. There are a variety of health benefits associated with alpinetin, including antibacterial, antioxidant, anticancer, antithrombotic, hypotensive, hypolipidemic, hypoglycemic, antiemetic, and analgesic properties (Wikan et al., 2022). Alpinetin has been shown in earlier investigations to have protective properties against acute lung damage and mastitis in mice (Chen et al., 2013). A study found that alpinetin significantly reduced TNF-α and IL-1β production in mice, MPO activity, diarrhea, colonic shortening, and histological damage. *In vitro*, alpinetin significantly reduced the production of proinflammatory cytokines TNF-α and IL-1β induced by NF-κB and NLRP3 inflammasomes (He et al., 2016).

#### 5.2.7 Procyanidin

Procyanidin is a flavonoid found primarily in green tea, grape skin, and grape seeds. It has been demonstrated that procyanidin absorbs oxygen radicals at a rate that is substantially greater than that of vitamin C and vitamin E (Ariga, 2004). Additionally, procyanidin has been shown to treat a wide range of inflammatory conditions (Pallarès et al., 2013). Procyanidin inhibited DSS-induced acute colitis in C57BL/6 mice by downregulating matrix metalloproteinases 9 expression, inhibiting the NF-κB signaling pathway, limiting NLRP3 inflammasome formation, and decreasing macrophage IL-1β levels (Chen et al., 2017).

#### 5.2.8 Kaempferol (Kae)

Kae (C_15_H_10_O_6_) is the primary active ingredient of many therapeutic plants. Additionally, Kae has antitumor and antioxidant properties in addition to its capacity to facilitate neurological recovery (Kumar, 2020). As well as reducing inflammation in mice caused by LPS, Kae is also capable of reducing HMGB1/TLR4 pathway-induced inflammation (Rajendran et al., 2014). Furthermore, Kae inhibits advanced glycation end products, which are responsible for NF-κB and NADPH oxidase expression. Also, Kae may reduce LPS/ATP-induced inflammation in cardiac fibroblasts by lowering the release of inflammatory proteins such as IL-6, IL-1β, and TNF-α(Hung et al., 2017). An additional clinical trial showed lower serum levels of C reactive protein, TNF-α, and IL-6 in type-2 diabetics who ate a Kae-rich diet. Kae-rich diets also reduced the generation of inflammatory mediators, leading to wound healing in mice with colitis. Kae modulated immune function in UC animal models by modifying the gut microbiota and different metabolites. Also, Kae reduced the activation of TLR4 to NF-κB induced by LPS *in vitro* (Qu et al., 2021).

### 5.3 Terpenoids

Terpenoids have a general formula of (C_5_H_8_) _*n*_, an oxygen-containing compound with varying levels of saturation (Christianson, 2017). They can be seen as a class of natural compounds linked by isoprene or isopentane in various ways. Terpenoids are widely found in nature, including higher plants, fungi, microorganisms, insects, and marine organisms. Terpenoids are an imperative class of compounds in Chinese herbal medicines. They are also an important natural spice and an indispensable raw material for the cosmetics and food industries (Masyita et al., 2022). According to the structure, terpenoids can be divided into monoterpenes, sesquiterpenes, diterpenes, triterpenoids, and polyterpenes. In addition to their biological effects, terpenoids also have a variety of physiological effects, including expectorant, cough, wind, sweating, insect repellent, analgesic, and so on (Table 3).

**TABLE 3:** Natural products of Terpenoids modulate NLRP3 protection in UC.

Natural products	Terpenoids				
Ingredient/Dose	Paeoniflorin 20 mg/kg; 10, 30, 100 and 300 μM	Celastrol 2 mg/kg	Asiatic Acid 10,30 mg/kg; 15, 30, 60 μM	Carnosic acid 50 and 100 mg/kg	Libertellenone M 10,20 mg/kg; 0.1,1,10 μM
Chemical name		3-Hydroxy-9β,13α-dimethyl-2-oxo-24,25,26-trinoroleana-1 (10),3,5,7-tetraen-29-oic acid		11,12-Dihydroxyabieta-8,11,13-trien-20-oic acid	
Chemical formula	C_23_H_28_O_11_	C_29_H_38_O_4_	C_30_H_48_O_5_	C_20_H_28_O_4_	
Type of cells	LPS-induced RAW264.7 cells		LPS-induced THP-1 cells		LPS-induced THP-1 and RAW 264.7 cells
Model of UC	DSS-induced UC mice	DSS-induced UC mice	DSS-induced UC mice	DSS-induced UC mice	DSS-induced UC mice
Route of administration	Oral	Oral	Oral	Oral	Intraperitoneal injection
Mechanism	IL-6, IL-1β and TNF-α↓; IL-10↑; NF-κB signaling↓	NLRP3↓; IL-23/IL-17 pathway↓; oxidative stress↓	TNF-α, IL-1β, IL-6 and IFN-γ↓; NLRP3, IL-1β and caspase-1↓	TNF-α, IL-17A, IL-6, IFN-γ, IL-1β and IL-18↓; NLRP3, IL-1β and caspase-1↓	NLRP3, IL-1β and IL-18↓; NF-κB signaling↓
References	Li et al. (2021)	Shaker et al. (2014)	Guo et al. (2015)	Yang et al. (2017a)	Fan et al. (2020b)

#### 5.3.1 Paeoniflorin (PF)

PF(C_23_H_28_O_11_), a monoterpene glycoside, is derived from the radices of *Paeoniae lactiflora Pall*, which have been used extensively in China for medicinal purposes for around 1,200 years (Sun et al., 2012; Yu et al., 2019). PF serves as an anti-cancer and anti-inflammatory agent (Lu et al., 2014), and clinical trials using PF or total glucosides of paeony have shown that PF can reduce the symptoms of IBD and inflammatory illnesses (Zhou et al., 2020). Reports indicate that PF prevents chemically induced colitis by reducing inflammation (Zhang et al., 2014)and modifying gut microbial metabolism (Fan et al., 2020). In Chinese medicine, the roots of plants in the *Paeonia genus*, including *Paeonia lactiflora Pall*., have been used to treat conditions similar to UC. Researchers examined the therapeutic effects of PF on mice with DSS-induced colitis discovering that PF had a preventative effect on colitis. With the PF treatment, activation of the NF-κB pathway was suppressed, reducing pro-inflammatory factor expression (Li et al., 2021).

#### 5.3.2 Celastrol

The root extracts of the three-wingnut and thunder god vine, *Tripterygium regelii*, contain a substance called celastrol (tripterine) Celastrol (C_29_H_38_O_4_) belongs to the quinone methides family of *Pentacyclic Nortriterpene Quinones* (Corson and Crews, 2007; Shi et al., 2020). The research shows that treatment with celastrol attenuated DSS-induced colon shortening and neutrophil infiltration. Besides, celastrol ameliorated DSS-induced colon injury and inflammatory signs as visualized by histopathology (Shaker et al., 2014). In the mouse model of DSS-induced colitis, celastrol prevents intestinal epithelial homeostasis loss by ameliorating acute intestinal injury. NLRP3 inflammasome, IL-23/IL-17 pathway, inflammatory cytokines, neutrophil infiltration, and E-cadherin expression are all inhibited by CSR, which suppresses colonic oxidative stress (Shaker et al., 2014; Jia et al., 2015).

#### 5.3.3 Asiatic acid

Asiatic acid (C_30_H_48_O_5_) is a natural triterpenoid molecule found in *Centella Asiatica* and *Terminalia catappa*, which is used in both Indian Ayurvedic and Chinese medicine (Tang et al., 2006; Huang et al., 2011). *C. asiatica* extract is usually used for medicine, tea, or aesthetic purposes (Bylka et al., 2013). In previous studies, asiatic acid has been demonstrated to have anti-inflammatory, neuroprotective, antioxidant, and anti-cancer properties (Gao et al., 2004; Zhang et al., 2012). Studies have shown that asiatic acid targets the NLRP3 inflammasome to treat UC(Guo et al., 2015). In this study, DSS-induced UC in mice significantly reduced the symptoms of colitis. Its mechanism involves inhibiting the ROS-NLRP3-caspase-1-IL-1β cascade in macrophages.

#### 5.3.4 Carnosic acid (CA)

CA (C_20_H_28_O_4_) is an alkane diphenol diterpene found in rosemary, therapeutic sage, and other plants. Rosemary extract has two main active ingredients, CA and carnosol, which are antioxidants and preservatives. It has been reported that 300 μg/mL rosemary leaf extract (89 mg/g carnosic acid) can inhibit the level of reactive oxygen species in the liver, brain, and stomach of rats and the lipid peroxidation products induced by sodium ferricyanide (Amaral et al., 2019). Studies have shown that CA in DSS-induced UC mice significantly prevented weight loss and colon length shortening in colitis improved the obvious infiltration of immune cells and the loss of crypt structure and goblet cells and explored its mechanism. It was found that CA significantly reduced MPO activity and F4/80^+^ macrophage infiltration in colon tissue, reduced the activation of p65 and c-Jun signals, and reduced caspase 1 activity to induce NLRP3 inflammasome activation (Yang et al., 2017).

#### 5.3.5 Libertellenone M (Lib M)

Lib M is the first diterpene of the pimarane type isolated from the marine fungus *Stibella fimetaris*. In plants, fungi, and marine animals, pimarane diterpenes are important tricyclic diterpenes (Kildgaard et al., 2017). A variety of biological functions are demonstrated by them, including anti-inflammatory properties (Costantino et al., 2009). Studies have shown that Lib M has strong anti-inflammatory activity by inhibiting the nuclear translocation of NF-κB and the assembly of the NLRP3 inflammasome in activated macrophages. In this study, it was found that Lib M can effectively alleviate the symptoms of colitis in the DSS-induced UC mouse model, indicating that Lib M has a reasonable therapeutic prospect as a UC drug targeting NLRP3 (Fan et al., 2020).

### 5.4 Saponins

Saponins are natural surface-active glycosides found in herbs such as ginseng, astragalus, and Sanqi. Recent studies have demonstrated that saponins can act as powerful anti-inflammatory agents and regulate immune homeostasis, especially in digestive diseases involving intestinal inflammation (Dong et al., 2019).

#### 5.4.1 Ginsenoside

Ginsenoside is a sterol compound, a triterpenoid saponin. It is only found in ginseng plants. Ginsenosides are regarded as the active ingredients in ginseng and thus become the target of research. Because ginsenosides affect multiple metabolic pathways, their efficacy is also complex, and the efficacy of various ginsenosides is difficult to isolate (Table 4).

**TABLE 4:** Natural products of Saponins, Polysaccharide, and others modulate NLRP3 protection in UC.

Natural products	Saponins			Polysaccharide		Others
Ingredient/Dose	Ginsenoside Rd 10, 20, 40 mg/kg; 5,10,20 μM	Ginsenoside Rk3 20, 40, 60 mg/kg	Dioscin 40 mg/kg	Pectic Polysaccharide 250 and 500 mg/kg; 320, 640 μg/mL	Astragalus Polysaccharide 100 and 200 mg/kg	Sinapic Acid 10 and 20 mg/kg
Chemical name						3,5-dimethoxy-4-hydroxycinnamic acid
Chemical formula	C_48_H_82_O_18_	C_36_H_60_O_8_	C_45_H_72_O_16_		C_10_H_7_ClN_2_O_2_S	C_11_H_12_O_5_
Type of cells	LPS-induced THP-1 cells			LPS-induced THP-1 cells		
Model of UC	DSS-induced UC mice	DSS-induced UC mice	DSS-induced UC mice	DSS-induced UC mice	DSS-induced UC mice	DSS-induced UC mice
Route of administration	Oral	Oral	Oral	Oral	Oral	Oral
Mechanism	IL- 1β, TNF-α and IL-6↓; NLRP3. caspase-1,ASC↓; P62↓	TNF-α, IL-1β and IL-6↓; NLRP3, ASC, and Caspase-1↓	ZO-1, Occludin↑; IL-10↑; TNF-α, IL-1β↓	IL-10↑; TNF-α, IL-6, MPO, Gal-3↓; NLRP3, ASC, and Caspase-1↓	NLRP3, ASC, and Caspase-1↓; IL-1β,IL-18↓	TNF-α, IL-1β, IL-6, IL-17α, IL-18, and IFN-γ↓; IL-1β, IL-18, ASC, NLRP3, caspase-1↓; IL-10, IL-4↑; Claudin-1, Occludin, and ZO-1 ↑
References	Liu et al. (2018a)	Tian et al. (2020)	Cai et al. (2021)	Pan et al. (2022)	Tian et al. (2017)	Qian et al. (2020)

##### 5.4.1.1 Ginsenoside Rd (G-Rd)

Many ginsenosides have been isolated and identified. Research has shown that ginseng can improve vitality, prolong life, and treat cardiovascular disease, depression, diabetes, malignant disease, depression, diabetes, malignant tumors, lung disease, and ulcers (Wang et al., 2012). Previous studies have shown that G-Rd (C48H82O18) has neuroprotective and anti-inflammatory effects, mostly by inhibiting the NF-κB signaling pathway (Bae et al., 2006). Studies have explored the effect of G-Rd on DSS-induced UC mice, indicating that G-Rd improves DSS-induced colitis. The mechanism is that G-Rd inhibits DSS-induced experimental colitis in mice by promoting the AMPK-ULK1-p62 axis-driven mitochondrial autophagy-mediated NLRP3 inflammasome inactivation and reducing macrophage IL-1β secretion (Liu et al., 2018).

##### 5.4.1.2 Ginsenosides Rg1-Rk3

G-Rg1 (C_42_H_72_O_14_) is the most abundant in ginsenosides and has a glucocorticoid-like structure. Hu et al. found that G-Rg1 downregulated the secretion of TNF-α, NO, the expression of iNOS and IBA-1 by inhibiting the activation of NF-κB and MAPK signaling pathways (Hu et al., 2011). G-Rg1 also inhibited the expression of iNOS, COX-2, TNF-α, and IL-1β through NF-κB in LPS-induced BV-2 microglia. It has been reported that G-Rg1 has anti-inflammatory activity *in vivo* in inflammatory animal models. In continuous observation *in vitro*, G-Rg1 effectively improved the symptoms of alcoholic hepatitis by inhibiting the activation of the NF-κB signaling pathway in TNBS-induced colitis animal models (Gao et al., 2015). Other studies have also shown that ginsenosides can accumulate in large quantities in the colon after oral administration in rats. They can be metabolized by various specific enzymes in the intestine (Lee, 2014).

Ginsenoside Rk3 (C_36_H_60_O_8_), which has a lower molecular weight, is produced by Rg1. Alcohol-induced liver injury can be suppressed by Rk3, as can kidney oxidative damage caused by cisplatin (Baek et al., 2017; Qu et al., 2019). A protective effect of ginsenoside Rk3 on DSS-induced colitis is achieved by inhibiting NLRP3 inflammasome expression and protecting intestinal barrier function (Tian et al., 2020).

#### 5.4.2 Dioscin

Dioscin (C_45_H_72_O_16_) is a natural steroidal saponin isolated and purified from the family Dioscoreaceae. Dioscin has excellent activity in metabolism, anticancer, anti-inflammation, and oxidative stress (Tao et al., 2018). Recent studies have found that dioscin can reduce serum uric acid levels and enhance uric acid excretion (Zhang et al., 2018). In addition, dioscin can improve atherosclerosis caused by hyperuricemia, inhibit the activation of the NF-κB signaling pathway, and the production of inflammatory cytokines to reduce the inflammatory response of gouty arthritis (Han et al., 2021). In UC-related experiments, studies have explored the role of Dioscin in DSS-induced UC. Dioscin can be effective in inhibiting DSS-induced colitis NF-κB, MAPK signaling, and NLRP3 pathway (Cai et al., 2021). These results suggest that Dioscin is a candidate drug for future treatment of UC.

### 5.5 Polysaccharide

An abundant polymer in nature, the polysaccharide is composed of many monosaccharide molecules linked together by glycosidic bonds. Plants and animals, as well as microorganisms, contain large amounts of it in their cell walls. It is known for its excellent safety and low toxicity, as well as its significant impact on the body. Researchers have found that polysaccharides can reduce insulin resistance, reduce blood sugar and cholesterol levels, and modulate the immune system (Table 4).

#### 5.5.1 Pectic polysaccharide

Pectic polysaccharides, a class of heteropolysaccharides rich in 1,4-galacturonic acid, are mostly composed of galactose, galactose, arabinose, and rhamnose. A study found that pectic polysaccharides prevented UC in mice (Fan et al., 2020; Chen et al., 2022). Traditional Chinese medicine Smilax China L. has been found to contain a new type of pectin polysaccharide SCLP3-2. It was found that SCLP had anti-inflammatory properties *in vitro* (Zhang et al., 2019). Researchers have demonstrated that SCLP can effectively improve DSS-induced acute UC by binding to Gal-3 and inhibiting its activity, thus blocking NLRP3 inflammasome signaling, indicating that SCLP may be an effective candidate drug to prevent and treat UC(Pan et al., 2022).

#### 5.5.2 Astragalus polysaccharide (APS)

APS (C_10_H_7_ClN_2_O_2_S) is a major component of *Astragalus membranaceus*, widely used in Chinese medicine and clinical research. *Astragalus* polysaccharide has immunomodulatory, anti-tumor, anti-lipid, anti-heart failure, and antioxidant effects (Cho and Leung, 2007; Lee et al., 2007). A TNBS-induced rat model of colitis was substantially reduced by APS through its control of cytokine expression, according to studies (Yang et al., 2014). Furthermore, *Astragalus* and *Lycium barbarum L.* (*Solanaceae*) polysaccharides help the intestinal barrier heal and protect against experimental UC(Zhao et al., 2014). APS can also downregulate the expression of NLRP3, caspase-1, and ASC in colon tissue, prevent the activation of the NLRP3 inflammasome, thereby reducing the expression of IL-18 and IL-1β and alleviating DSS-induced colon inflammation (Tian et al., 2017).

### 5.6 Others

#### 5.6.1 Sinapic acid

Sinapic acid (C_11_H_12_O_5_) is a naturally occurring hydroxycinnamic acid commonly found in fruits, vegetables, grains, and oilseed crops. It is also found in wine and vinegar (Chen and longevity, 2016). Sinapic acid exhibits a variety of pharmacological effects, including antibacterial, anticancer, anti-inflammatory, anticancer, antimutagenic, antiglycemic, and antioxidant properties. A study by Lee has shown that TNBS-induced colitis is capable of reducing inflammation in mice (Lee, 2018). Sinapic acid also reduced the signs of DSS-induced colitis in mice by controlling NLRP3 inflammasome. DSS-induced mice treated with Sinapic acid had significantly decreased levels of NLRP3, IL-1b, ASC, and Caspase-1, which improved symptoms of colitis (Qian et al., 2020).. (Table 4)

## 6 Prospects

UC is an inflammatory disease of the colon that is non-specific. As is well known, UC is closely related to the inflammatory response. In recent years, research on the mechanisms and targeting of NLRP3 in UC has become progressively advanced. Research on the activation and regulation of NLRP3 inflammasome is mostly focused on immune cells and animals, but clinical targeting of NLRP3 inflammasome has also progressed. Clinically, UC is treated by inhibiting NLRP3 activation through antibodies or antagonists, including the IL-1β receptor blocker Rilonacept, the IL-18 blocker GSK1070806, the NLRP3-related ATPase inhibitor Bay 11-7082, and the caspase-1 inhibitor Eugenolide. As NLRP3 is a core component of the inflammatory response, if the relationship between NLRP3 and intestinal microbes and inflammation-related factors can be analyzed for its related inflammatory signaling pathways, its expression status and specific mechanisms in the course of IBD can be elucidated and further considered for clinical diagnosis and treatment of UC, it is expected that lead compounds targeting NLRP3 inflammasome can be developed to treat UC. The active ingredients of Chinese medicine have made great progress in the clinical treatment of UC, and related studies have attracted much attention. The main therapeutic mechanisms include inhibition of signaling pathways such as NF-κB, restoration of Th17/Treg imbalance, regulation of inflammatory factor expression, and slowing down inflammation.

A variety of active components of traditional Chinese medicine, including flavonoids, polysaccharides, polyphenols, terpenoids, and saponins, can protect against UC-related colitis symptoms by regulating NLRP3-related pathways. The mechanism of action of these compounds mainly involves the regulation of NLRP3 to downregulate the expression of inflammatory factors to reduce the inflammatory response and protect against tissue damage. NLRP3 is the core link in the inflammatory response. Natural products can regulate the relationship between NLRP3 and intestinal microorganisms and inflammation-related factors according to their related inflammatory signaling pathways. At present, it is more commonly used in clinical practice to combine traditional Chinese medicine compound preparations with traditional therapeutic drugs. Natural products have less clinical application in the treatment of UC and are still in the research and development stage, but the research on animal models has been more mature. Not only that, many natural products have been used in animal models of UC with comparable efficacy to therapeutic-positive drugs. Therapeutic strategies targeting NLRP3 have shown great potential. Although precise targeting NLRP3 therapeutic strategies are still in the research stage and many obstacles need to be overcome for their application in clinical treatment, it brings hope to the treatment of UC.

Chinese plant polysaccharides can regulate the imbalance of gut microbes, repair intestinal barrier damage, and alleviate intestinal inflammation through NLRP3. The flavonoids can inhibit inflammation, and regulate intestinal flora and their metabolites through NLRP3. The therapeutic effects of natural products of Chinese medicine are often multi-targeted, and how to correlate the targets of Chinese medicine with inflammasome deserves more research. In this respect, perhaps network pharmacology can provide some reference. With its holistic and multi-layered nature, the effective targets of herbal medicine in modulating inflammasome can be searched for and investigated in greater depth.
